# Foveal Phase Retardation Correlates With Optically Measured Henle Fiber Layer Thickness

**DOI:** 10.3389/fmed.2022.846738

**Published:** 2022-04-15

**Authors:** Phillip T. Yuhas, Marisa L. Ciamacca, Keith A. Ramsey, Danielle M. Mayne, Elizabeth A. Stern-Green, Matthew Ohr, Aaron Zimmerman, Andrew T. E. Hartwick, Dean A. VanNasdale

**Affiliations:** ^1^College of Optometry, The Ohio State University, Columbus, OH, United States; ^2^Department of Ophthalmology and Visual Sciences, College of Medicine, The Ohio State University, Columbus, OH, United States

**Keywords:** Henle fiber layer, scanning laser polarimetry, directional optical coherence tomography, fovea, macula HFL thickness, phase retardation

## Abstract

This study quantified and compared phase retardation distribution in the central macula with the thickness of the Henle fiber layer (HFL). A scanning laser polarimeter (SLP) was used to acquire 20° × 40° macular-centered images, either with fixed corneal compensation or with variable corneal compensation, in two cohorts of clinically normal subjects (*N* = 36). Phase retardation maps from SLP imaging were used to generate a macular cross pattern (fixed compensation) or an annulus pattern (variable compensation) centered on the macula. Intensity profiles in the phase retardation maps were produced using annular regions of interest at eccentricities from 0.25° to 3°. Pixel intensity was averaged at each eccentricity, acting as a surrogate for macular phase retardation. Directional OCT images were acquired in the horizontal and vertical meridians in all subjects, allowing visualization of the HFL thickness. HFL thickness was manually segmented in each meridian and averaged. In both cohorts, phase retardation and HFL thickness were highly correlated in the central 3° assessed, providing further evidence that the source of the phase retardation signal in the central macula is dominated by the HFL and that the center of the macula on cross sectional imaging corresponds closely with the center of the macular cross on SLP imaging.

## Introduction

The cone photoreceptors in the central macula have important roles in mediating high spatial acuity and color discrimination in the human visual system. The axons of these central photoreceptors can exceed 500 μm in length, which is considerably longer than the axons (~20 μm) of photoreceptors located at greater eccentricities (1, 2). As another difference, central cone axons are oriented relatively perpendicular to light incident on the retina, and the lateral displacement of their axon terminals produces a foveal pit that is devoid of inner retinal neurons. These bundles of cone axons are the primary contributors to the Henle fiber layer (HFL) present in the macular region of the primate retina.

Using traditional clinical imaging modalities, the HFL is difficult to assess and quantify. Cross-sectional optical coherence tomography (OCT) imaging in its routine use poorly delineates the HFL (3, 4). The HFL appears as an optically indistinct/transparent section of the outer retina and is difficult to differentiate from the outer nuclear layer. As a result, the HFL and the outer nuclear layer are commonly combined and assessed as a single entity in OCT imaging studies (5, 6).

Alternative imaging methods, specifically directional OCT imaging (7), enables improved differentiation and visualization of the Henle fiber layer. By aligning the incident light with the HFL's primary scattering angle, the reflectivity and the optical contrast of the HFL, relative to the outer nuclear layer, is enhanced. Lateral displacement of the imaging beam across the pupil allows visualization of the HFL as a hyper-reflective layer in the OCT cross section. This technique is directionally dependent, as the offset position (nasal vs. temporal; superior vs. inferior) of the beam at the pupil determines the relative emphasis of the HFL appearance on the two sides (distal vs. proximal) of the OCT image (7). Directional OCT imaging provides a visible landmark of the anterior border of the HFL on the side of the offset and the posterior border on the contralateral side of the offset. Combining two images from opposite offset entry positions can be used to quantify the HFL thickness along a common meridian. This technique has been validated and has been shown to be accurate (8) and to be repeatable across imaging sessions and across graders with little variability (9).

Scanning laser polarimetry (SLP) is an additional technique capable of quantifying the HFL based on the birefringent properties of the microtubules contained in the macular cone axons (10). Phase retardation, or meridional differences in index of refraction, is an optical signature of the HFL and can be used to localize the fovea in normal (11) and in diseased eyes (12), track eye position (13, 14), and quantify changes associated with normal aging (15) and with ocular disease (10, 16).

A key feature in SLP imaging of the posterior pole is the macular cross, an interaction between the corneal polarization properties (17) and those of the radially oriented HFL. The polarization properties of the cornea need to be accounted for when analyzing macular birefringence. Early-generation SLP devices were equipped with a fixed corneal compensator. Although this compensator is not ideal in the assessment of the fovea, point-by-point quantification of pixel intensity can be used to measure the amplitude of phase retardation in the macular cross along a circular region of interest at different eccentricities. This characteristic pattern exhibits a maximum phase retardation occurring within the central 3° in normal subjects, with reduced amplitudes associated with normal aging (15) and in patients with non-exudative age-related macular degeneration (16). A variable corneal compensator on newer SLP devices is able to more fully account for corneal birefringence and produces an annular pattern of birefringence around the retina. However, foveal phase retardation measurements made with full corneal compensation are less fully characterized than those made with fixed corneal compensation. Both corneal compensation techniques, fixed and variable, are used in this study.

While it has long been speculated that the foveal phase retardation signal originates from the HFL, a comparison with HFL assessments from alternative imaging techniques could test the validity of this assumed relationship. This study will examine two optical properties associated with the HFL using directional OCT and SLP in order to assess the correlation between the HFL thickness and phase retardation distribution at eccentricities from 0° to 3°, centered on the fovea. It is hypothesized that HFL thickness from directional OCT will correlate closely with the phase retardation distribution from SLP.

## Methods

This cross-sectional study followed the tenets of the Declaration of Helsinki and was approved by the Institutional Review Board in Biomedical Sciences at the Ohio State University. Informed consent was obtained from all subjects before data collection.

### Subjects

Two cohorts of healthy subjects were prospectively recruited from the Ohio State University. The first cohort comprised 11 adults (age range 25–54 years, 63% female). The second cohort comprised 25 healthy adults (age range 20–58 years, 52% female). All subjects received a dilated fundus examination within at least 1 year of participation and did not have a previous diagnosis of ocular disease. Spherical equivalent refractive error ranged between +2D and −8D for all participants.

### Protocol and Instrumentation

All subjects attended a single study session where images of the retina were acquired using both SLP and OCT. Prior to imaging, subjects underwent pupil dilation either with 1% tropicamide or with a combination of 1% tropicamide and 2.5% phenylephrine to achieve maximal pupil dilation, which is needed to offset the imaging beam appropriately during directional OCT imaging.

#### Directional Optical Coherence Tomography Imaging and Processing

**Figure 1:**
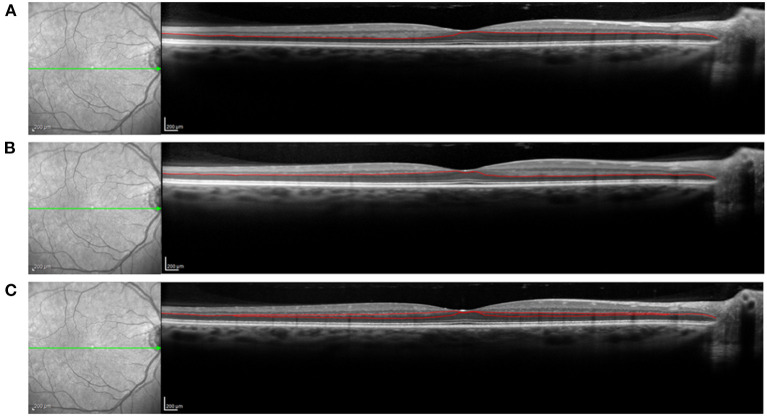
OCT imaging of the Henle fiber layer (HFL). Offset of the entry position of the imaging beam though the pupil results in improved visualization of the HFL. The posterior border of the HFL is more clearly delineated on the distal side of the pupil offset, while the anterior border is more clearly delineated on the proximal side of the pupil offset. **(A)** Nasal offset of the imaging beam through the pupil, resulting in improved visualization of the posterior border of the temporal HFL and anterior border of the nasal HFL with manual segmentation line. **(B)** Temporal offset of the OCT imaging beam through the pupil, resulting in improved visualization of the posterior border of the nasal HFL and anterior border of the temporal HFL with manual segmentation line. **(C)** No offset of OCT imaging beam through the pupil, with poor delineation of the HFL. Segmentation lines from nasal and temporal offsets are included to demonstrate the HFL thickness profile along the horizontal meridian. The green arrows on the en face images represent the meridian of interest.

All study participants sat for directional OCT imaging. Cross sectional OCT images were acquired from both eyes using a Heidelberg Spectralis spectral domain OCT (Heidelberg Engineering, Heidelberg, Germany). Vertical and horizontal 30° cross sectional images centered on the fovea were acquired for each subject. To minimize noise, each cross section used for analysis was the composite average of 100 individual registered b-scans acquired using the Heidelberg TruTrack Active Eye Tracking. Images were acquired at the high-resolution setting, which collects 1,536 a-scans per b-scan. The nominal axial and lateral resolutions are 7 and 14 μm, respectively. During imaging, the compiling average of b-scans is shown while a live image is simultaneously displayed. The live image was used to ensure that the angular orientation of the retina remained constant throughout the collection of the 100 images used for each averaged cross section. Three cross sections were acquired in both the horizontal and vertical meridian, for a total of 6 images. One cross section was acquired by aligning the imaging beam to the optical axis passing through the center of the fovea, resulting in an image where retinal displacement is symmetric about the foveal center. Two additional images were acquired in each the horizontal and vertical meridians by offsetting the entrance of the imaging beam position by 3 mm on either side of the centered foveal optical axis position. Offsetting the entry position along a meridian enables visualization of the Henle fiber layer on the contralateral side of the offset, and minimizes visualization on the ipsilateral side (Figures 1A,B). The six collected images, all centered on the fovea, include a horizontal cross sectional image aligned with the foveal axis, a directional OCT cross section with a right lateral offset in the pupil, a directional OCT image with a left lateral offset in the pupil, a vertical cross sectional image aligned with the foveal axis, a directional OCT image with a superior offset in the pupil, and a directional OCT image with an inferior offset in the pupil.

Images from the right eye with a quality metric >20 dB were kept for analysis. The HFL was manually segmented from the cross sectional OCT images using Adobe Photoshop (Adobe, San Jose, CA). The posterior location of the Henle fiber layer on the distal side of each of the directional OCT images, and the anterior border was segmented on the proximal side. The anterior and posterior boundaries of each aligned directional OCT image were combined to generate the HFL thickness profile along that meridian (Figure 1C).

**Figure 2:**
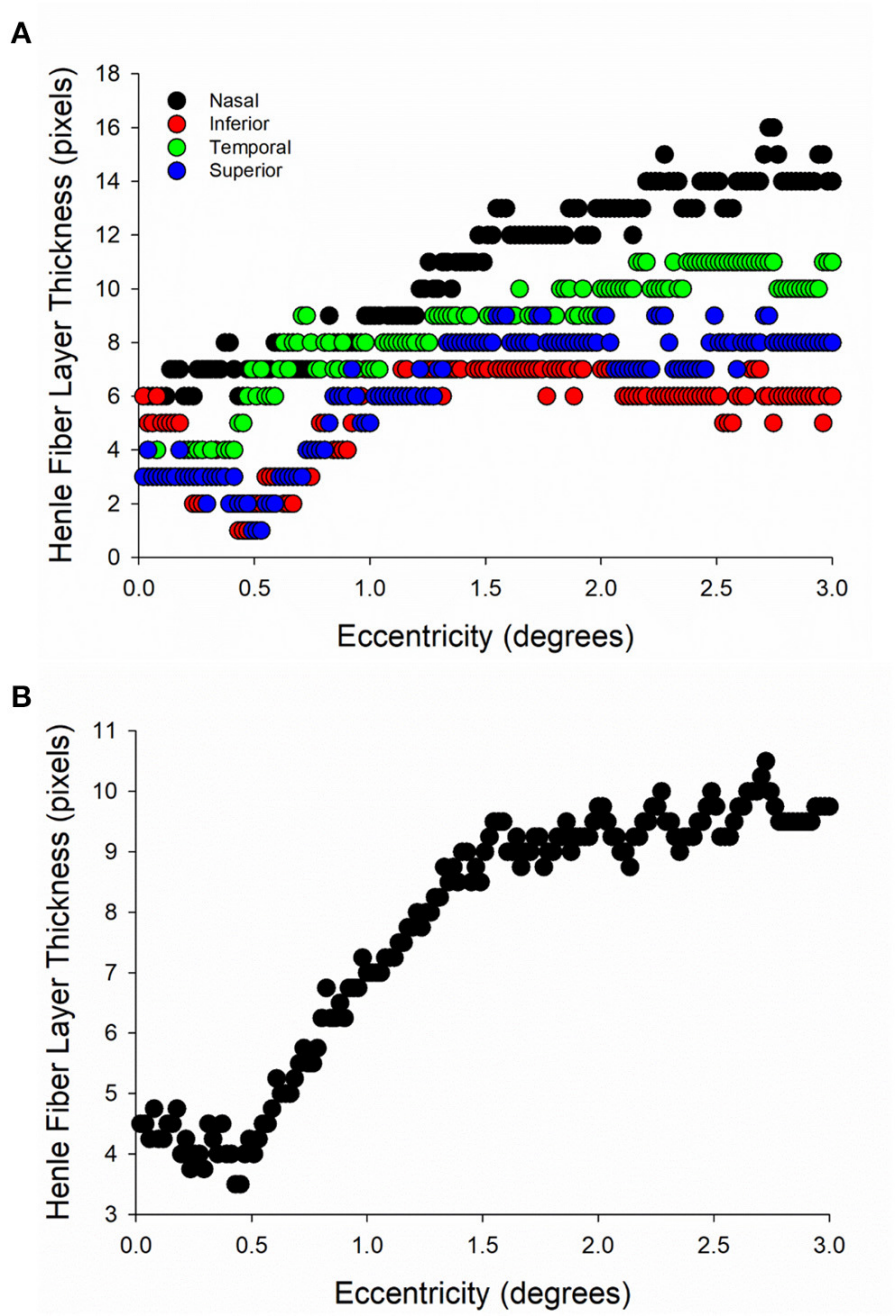
Henle fiber layer (HFL) thickness distribution in a single subject from directional OCT imaging. **(A)** HFL thickness in each of the 4 meridians of interest. **(B)** HFL thickness averaged across the four meridians in the same subject.

The foveal center was localized using the longest photoreceptor outer segment distance on the OCT cross sectional images in both the horizontal and vertical images. Custom MATLAB programming (Mathworks, Natick, MA) computed the thickness in pixels from the segmented HFL images centered on the foveal position, and Henle fiber layer thickness was measured for each of 4 meridians: temporal, superior, nasal, and inferior (Figure 2A). Because there is variability in the HFL thickness in different meridians (8), an average of the four meridians was calculated and used for analysis (Figure 2B). HFL thickness was then measured at 0.25° intervals from 0° to 3° eccentricity from the center of the fovea. Axial length was not measured as part of this study, which prevented the correction of lateral magnification. Instead, a conversation factor of 320 microns/degree was used to identify measurement locations on the OCT image. This approach is consistent with previous work that compared between OCT images and SLP images (18).

#### Scanning Laser Polarimetry Imaging and Processing

**Figure 3:**
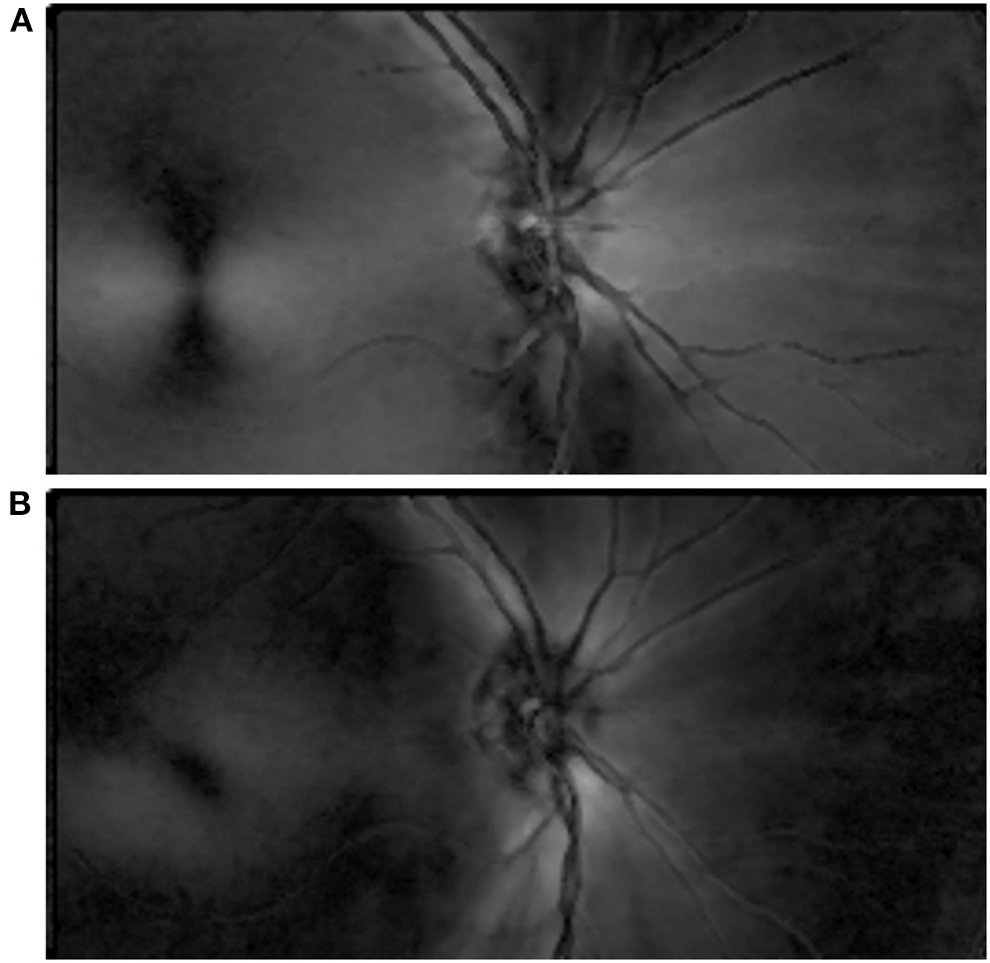
Phase retardation distribution map in 20° × 40° scanning laser polarimetry image. **(A)** An image acquired from a subject in the first cohort included a fixed polarization element that partially compensated for corneal birefringence. Due to incomplete compensation, a characteristic macular cross pattern resulted, centered on the macula. **(B)** An image acquired from a subject in the second cohort included a variable polarization element that more fully accounted for corneal birefringence than a fixed-compensation setting. It manifested an annulus, centered on the macula.

All subjects then sat for confocal SLP imaging using a GDx device (Laser Diagnostic Technologies, San Diego, CA). A 20° vertical × 40° horizontal macular-centered image was collected from both eyes of each subject using a raster scanning 780 nm polarized light source on the retina. The GDx instrument used to image the first cohort had a fixed birefringent element with a magnitude of 60 nm (single pass retardance) and a slow axis oriented at 15° nasally downward to compensate for corneal birefringence. Fixed compensation in this instrument does not result in complete corneal compensation, however, and results in a radial macular cross pattern (Figure 3A), instead of the annulus pattern expected to occur with full compensation (19). The second cohort was imaged with the same SLP device but utilized variable corneal compensation, which more fully corrects for corneal birefringence than fixed compensation. This approach reduces or eliminates the cross pattern in the macula that is characteristic of fixed compensation and allows for more direct annular assessment of the HFL (Figure 3B). Images were kept for analysis if the machine-generated quality score was ≥8, per the recommendation of the manufacturer.

**Figure 4:**
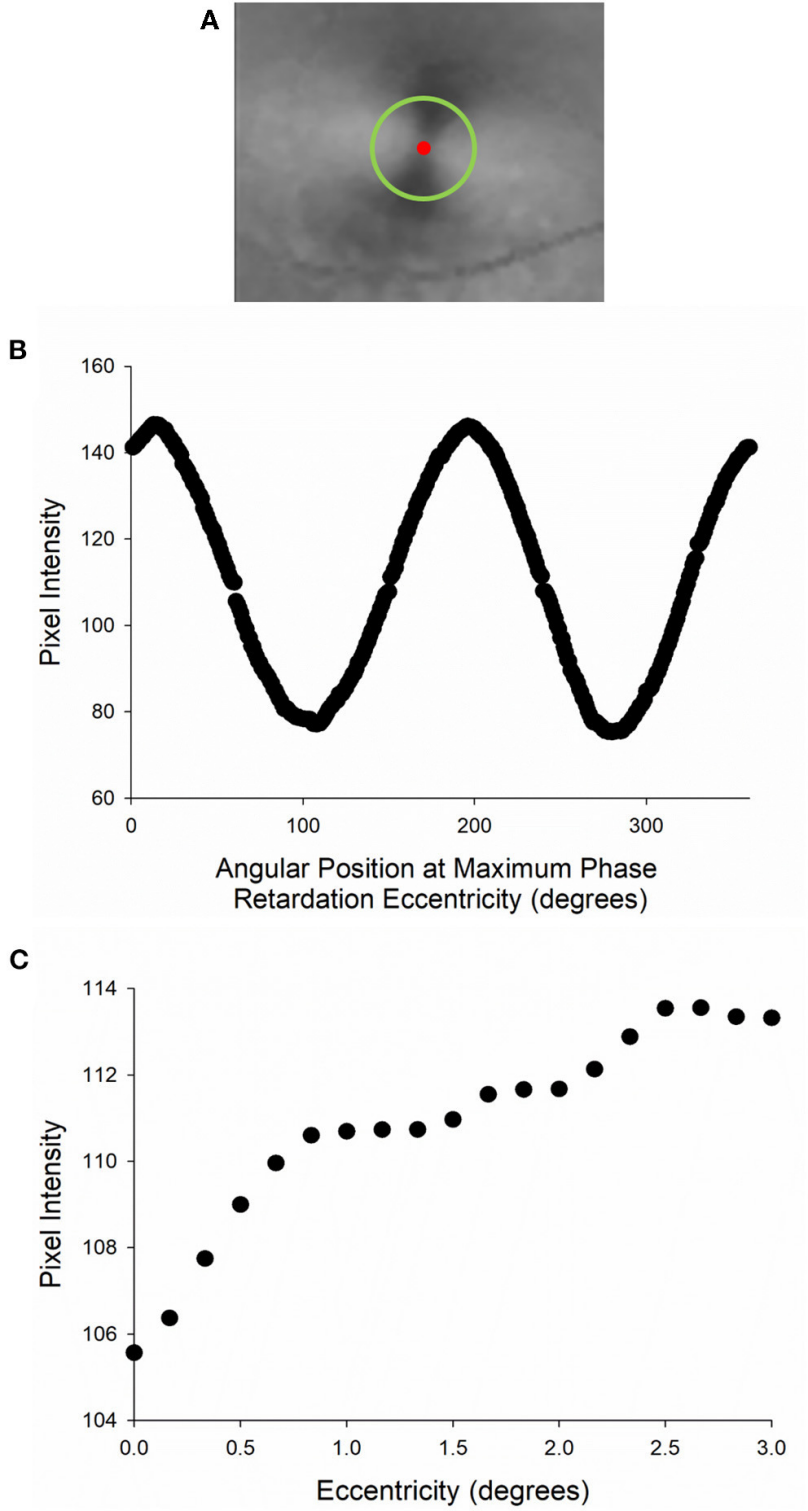
Analysis of phase retardation distribution map. **(A)** Center of macular cross (red circle) with a circular region of interest (green circle). **(B)** Pixel intensity profile along the circular region of interest. **(C)** The amplitude of pixel intensity was used as a relative measure of phase retardation and plotted as a function of eccentricity.

The phase retardation maps of the right eye that include the macular cross (fixed corneal compensation) or annulus (variable corneal compensation) were exported from the GDx and imported into MATLAB for pixel-intensity analysis. In summary, concentric rings at varying eccentricities from the fovea produced pixel intensity profile curves that exhibit either a sinusoidal pattern at double the frequency of the circular region of interest if imaged with fixed compensation (Figures 4A,B) or a linear pattern if imaged with variable compensation. For all images, pixel intensity was then averaged across all points contained within eccentric rings centered on the fovea from 0° to 3° (Figure 4C). The average pixel intensity represented the phase retardation signal across each eccentric ring.

### Statistical Analysis

All statistical analyses were conducted with SigmaPlot 14.5 (Systat, Palo Alto, CA).

#### Comparison of Henle Fiber Layer Thickness

The thickness of the HFL across all eccentricities was compared between the first cohort and the second cohort. Since the data were not normally distributed, a Mann-Whitney Rank Sum Test (α = 0.05) compared the thickness between the two cohorts.

#### Comparison of Pixel Intensity Amplitude

The pixel intensity measured by SLP across all eccentricities was compared between the first cohort (fixed corneal compensation) and the second cohort (variable corneal compensation). Since the data were not normally distributed, a Mann-Whitney Rank Sum Test (α = 0.05) compared the intensity between the two cohorts.

#### Comparison of Pixel Intensity Variability

The variability of pixel intensity measurements made with the fixed-compensated SLP (first cohort) was compared to the variability of the pixel intensity measurements made with the variable-compensated SLP (second cohort). For both instruments, the coefficient of variation was measured at each eccentric ring, from 0° to 3° in 0.25° intervals. These values were then averaged for an overall measurement of variability for each cohort. A Mann-Whitney Rank Sum Test (α = 0.05) compared the variability between the two cohorts.

#### Correlation Between Henle Fiber Layer Thickness and Pixel Intensity

**Figure 5:**
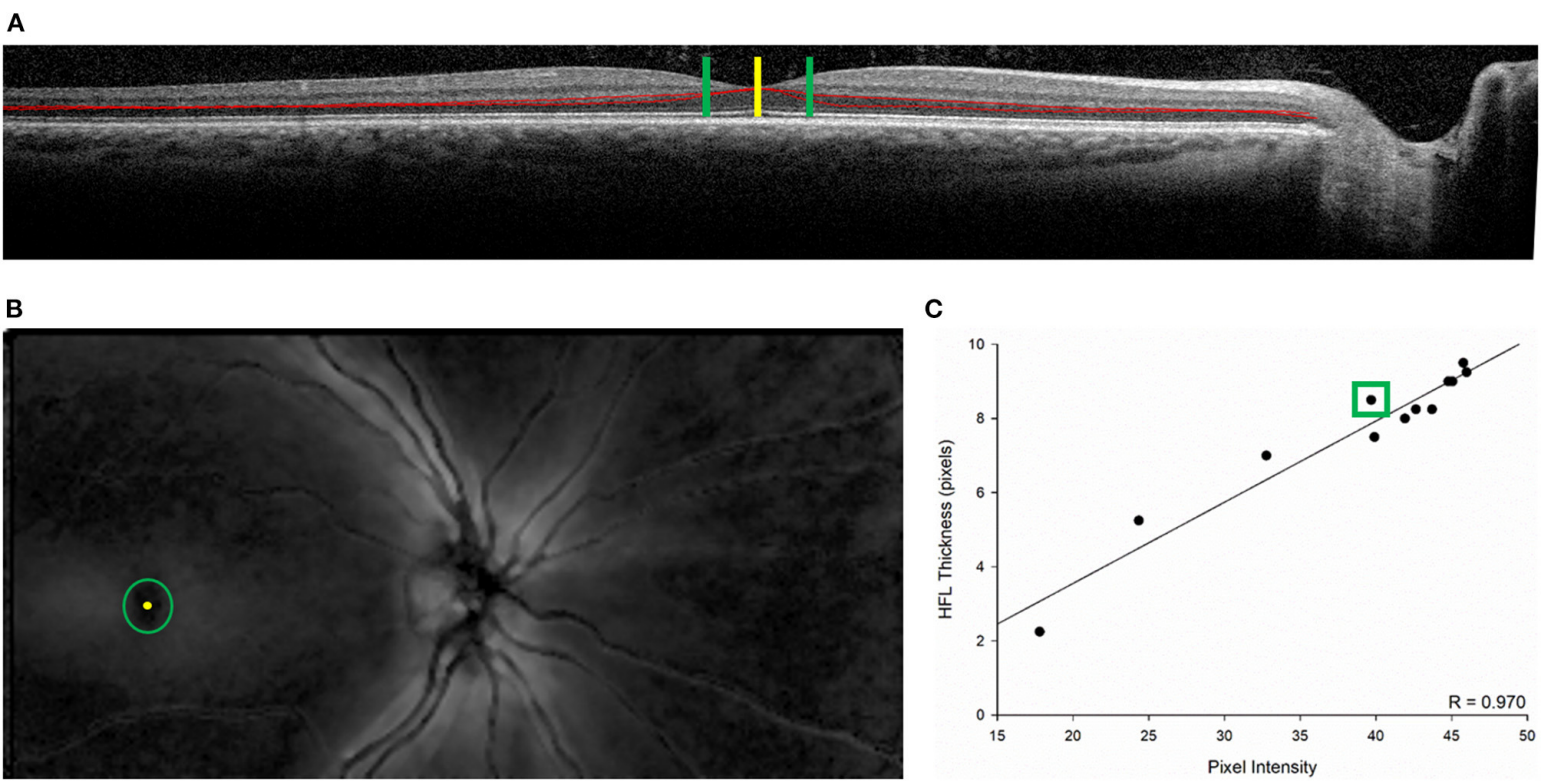
Example of the association between Henle fiber layer (HFL) thickness and phase retardation in an individual subject from the second cohort. **(A)** Directional optical coherence tomography image along the horizontal axis with the HFL outlined in red. The yellow vertical bar represents the center of the fovea. The green vertical bars mark 1° of eccentricity (temporal on the left and nasal on the right) from the center of the fovea. **(B)** Scanning laser polarimetry image of the posterior pole. Bright regions within the image indicate high phase retardation caused by tissue birefringence. The yellow circle marks the center of the fovea. The green ring indicates 1° of eccentricity from the center of the fovea. **(C)** Regression plot showing the association between pixel intensity (a proxy for magnitude of phase retardation) and HFL thickness. The data point in the green box represents (i, y-axis) HFL thickness averaged from the superior, inferior, nasal (right green bar in **A**), and temporal (left green bar in **A**) meridians 1° from the central fovea and (ii, x-axis) phase pixel intensity averaged along an eccentric ring (green ring in **B**) 1° from the central fovea. The other data points represent the other eccentricities measured (0°-3° eccentricity in 0.25° steps from the center of the fovea).

For all subjects, the HFL thickness, as measured using directional OCT imaging, and pixel intensity, a representation of the phase retardation signal, as measured with SLP, were plotted against each other at 0.25° intervals from 0° to 3° eccentricity (Figure 5). The correlation coefficients (R) for the association between the HFL thickness and pixel intensity at eccentricities between 0° and 3° in 0.25° increments were determined for each subject. Individual R values were then converted to *z*-values using Fisher-Z-Transformation. *Z*-values were then averaged within each cohort and transformed back to *R*-values to generate a representative *R* and *R*^2^-values for the cohorts.

## Results

### Henle Fiber Layer Thickness Measured by Directional Optical Coherence Tomography

**Figure 6:**
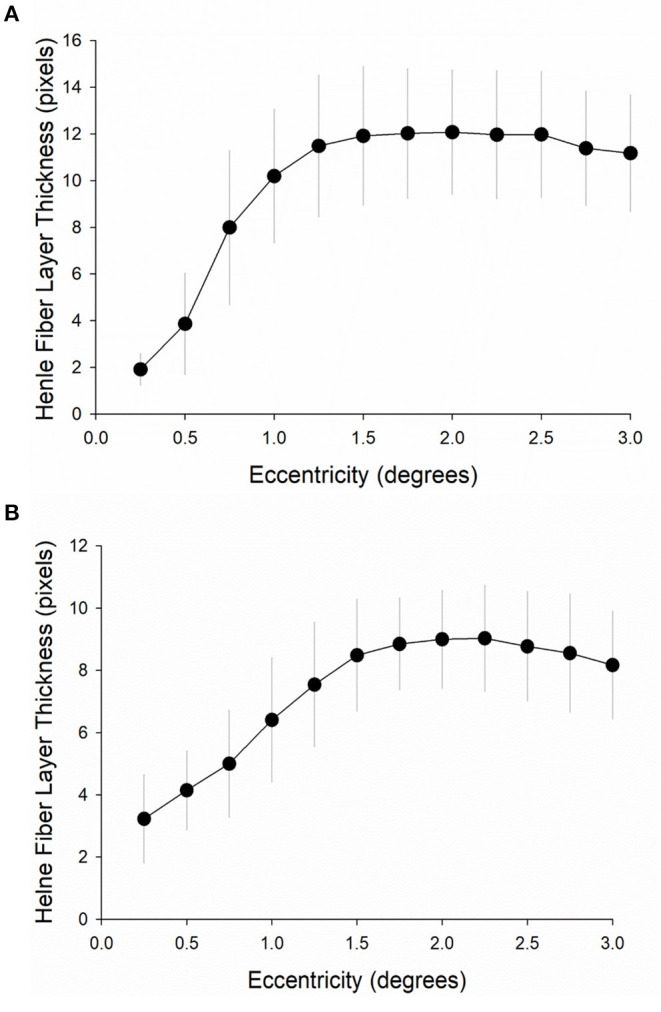
Henle fiber layer thickness at increasing macular eccentricities. Henle fiber layer thickness was measured at 12 different macular eccentricities from 0° to 3° from the foveal center in 0.25° steps **(A)** in the first cohort (*n* = 11) and **(B)** in the second cohort (*n* = 22). Error bars are one standard deviation from the mean (circles).

Three subjects in the second cohort had at least one image with a quality score ≤20 dB (for representative image, see Supplementary Figure 1); these subjects were removed from analysis. For both the first cohort (Figure 6A) and for the second cohort (Figure 6B), the HFL thickness increased as the retinal eccentricity changed from 0° to 2° before decreasing at greater macular eccentricities. Across all eccentricities, the first cohort (median HFL thickness [25% quartile, 75% quartile] = 11.4 [8.55, 12.0] pixels) had a significantly (*n* = 0.015, Mann-Whitney Rank Sum Test) thicker measured HFL than the second cohort (8.33 [5.35, 8.83] pixels).

### Pixel Intensity Measured by Scanning Laser Polarimetry

#### Signal Pattern and Inter-cohort Differences in Amplitude

**Figure 7:**
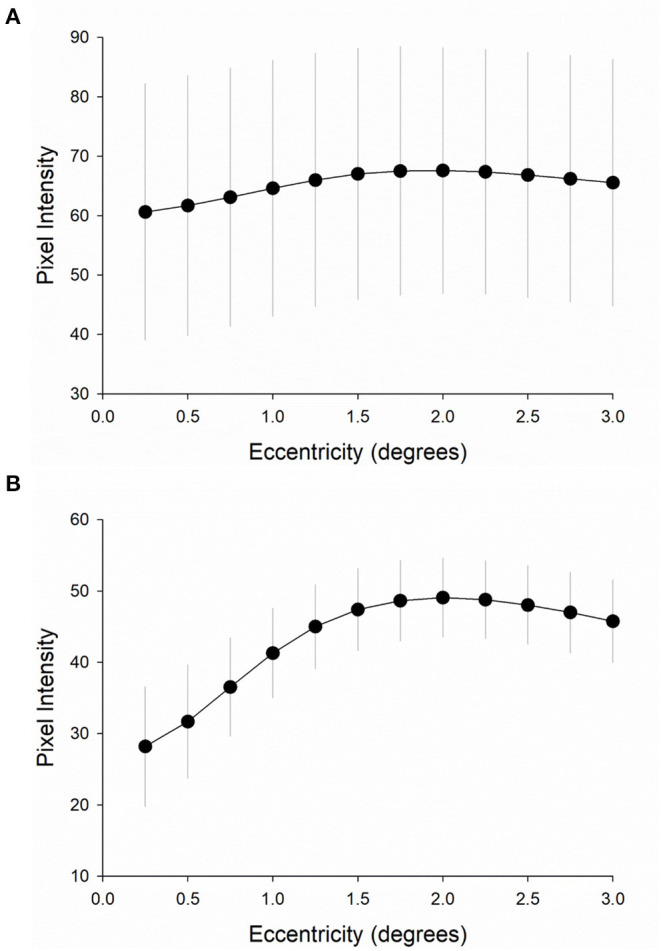
Pixel intensity at increasing macular eccentricities. Pixel intensity was measured at 12 different macular eccentricities (from 0° to 3° from the foveal center in 0.25° steps) **(A)** in the first cohort (fixed-compensation scanning laser polarimetry, *n* = 11) and **(B)** in the second cohort (variable-compensated scanning laser polarimetry, *n* = 25). Error bars are one standard deviation from the mean (circles).

All SLP scans had a quality score ≥8 and were therefore analyzed. For both the first cohort (fixed-compensated SLP; Figure 7A) and for the second cohort (variable-compensated SLP; Figure 7B), pixel intensity increased as the retinal eccentricity increased from 0 to 2 degrees before decreasing at greater retinal eccentricities. Across all eccentricities, pixel intensity generated by the fixed-compensated SLP (median pixel intensity [25% quartile, 75% quartile] = 66.1 [63.5, 67.3]) was significantly (*p* < 0.001, Mann-Whitney Rank Sum Test) higher than that generated by the variable-compensated SLP (46.4 [37.7, 48.5]).

#### Inter-cohort Variability

The first cohort (fixed-compensated SLP) and the second cohort (variable-compensated SLP) exhibited different magnitudes of variability. In both cohorts, the coefficients of variation near the center of the fovea were relatively large, and those at farther eccentricities were relatively small (Table 1). However, across all eccentricities, the first cohort exhibited significantly (*p* < 0.001, Mann-Whitney Rank Sum Test) more variability (median coefficient of variation [25% quartile, 75% quartile] = 31.5 [30.9, 34.1]) than the second cohort (12.4 [11.4, 17.9]).

**Table 1:** Variability of pixel intensity at increasing macular eccentricities.

**Eccentricity (degrees)**	**First cohort (*n* = 11)**	**Second cohort (*n* = 25)**
0.25	35.5	29.8
0.50	35.4	25.0
0.75	34.4	18.8
1.00	33.3	15.2
1.25	32.3	13.1
1.50	31.5	12.1
1.75	30.9	11.6
2.00	30.6	11.2
2.25	30.5	11.1
2.50	30.8	11.4
2.75	31.3	12.0
3.00	31.6	12.6

*Values are coefficients of variation. Phase retardation was measured with a scanning laser polarimeter with a fixed corneal compensator in the first cohort and with a variable corneal compensator in the second cohort*.

### Correlation Between Pixel Intensity and Henle Fiber Layer Thickness

**Figure 8:**
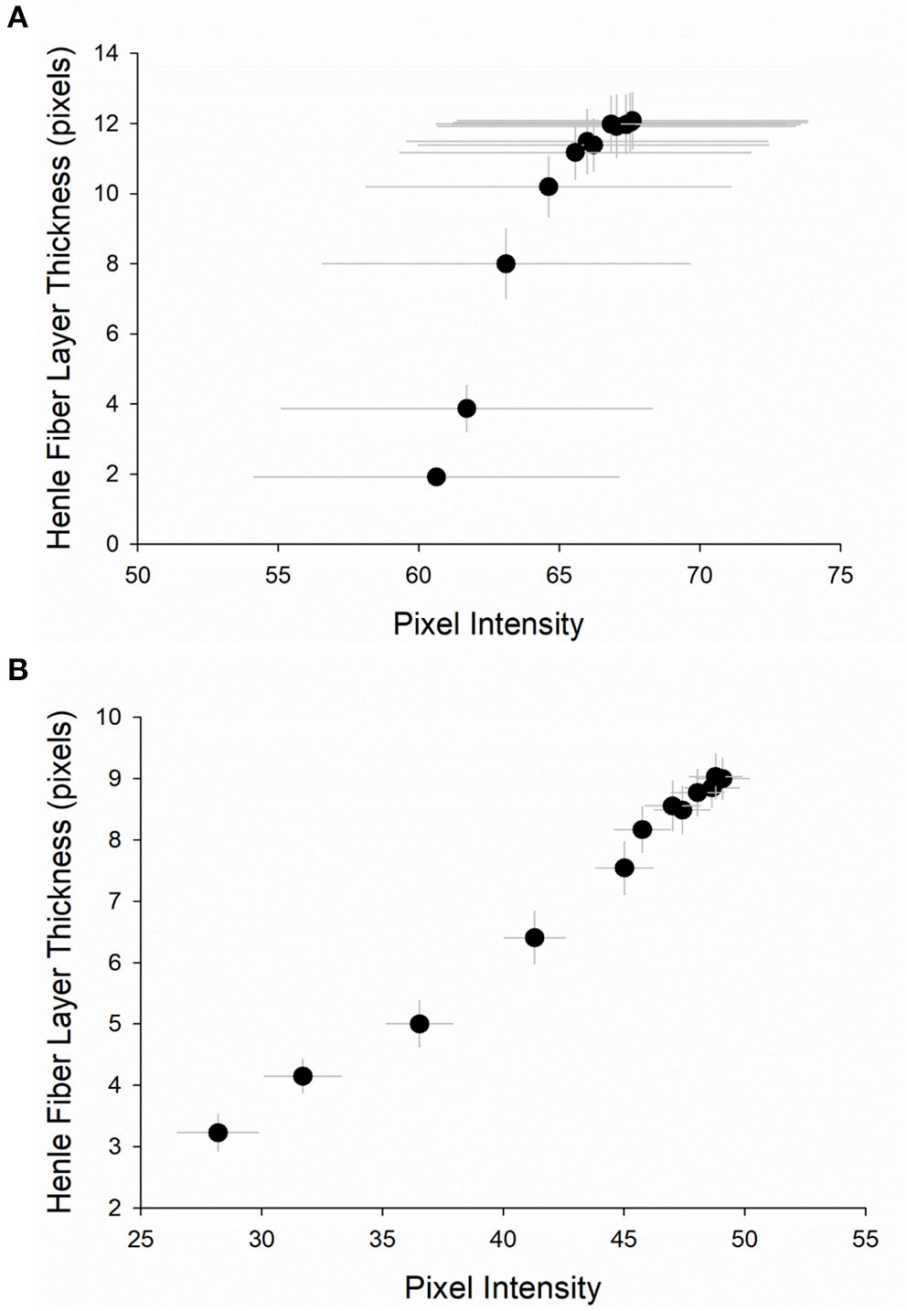
Representation of the relationship between pixel intensity and Henle fiber layer thickness **(A)** in the first cohort (*n* = 11) and **(B)** in the second cohort (*n* = 22). Circles are the mean at each of the 12 eccentricities, from 0° to 3° (0.25° steps) in the central macula. Vertical and horizontal error bars are the standard error of the mean.

There was a strong correlation between pixel intensity and HFL thickness in both cohorts. In the first cohort, the average *R*^2^-value was 0.785 (range: 0.031–0.988; Supplementary Figure 2). Figure 8A contains a representation of this correlation, using mean values for pixel intensity and for HFL thickness at all eccentricities. In the second cohort, the average *R*^2^-value was 0.872 (range: 0.043–0.974; Supplementary Figure 3). Figure 8B contains a representation of this correlation, using mean values for pixel intensity and for HFL thickness at all eccentricities. Combining subjects from the first and second cohorts, the overall *R*^2^-value was 0.846.

## Discussion

This study used two clinically available imaging modalities, OCT and SLP, to establish a correlation between HFL thickness and phase retardation in the central macula. In two healthy cohorts, HFL thickness increased from to foveal center to 2° eccentricity, and then decreased beyond 2°. This result is consistent with previous reports from clinical (8) and from histological (5) studies on normal populations. Foveal pixel intensity—a representation of tissue birefringence, with high pixel intensity indicating high phase retardation—was also measured. Similar to the HFL thickness results, in both cohorts pixel intensity increased from the foveal center to 2° eccentricity, beyond which it receded. There was a strong correlation between HFL thickness and pixel intensity across the central 3° of the macula. The fixed-compensated SLP device (used on the first cohort) manifested more variability in its pixel intensity measurements than the variable-compensated SLP device (used on the second cohort).

### Correlation Between Henle Fiber Layer Thickness and Phase Retardation

To the authors' knowledge, this is the first study to report a correlation between HFL thickness and phase retardation amplitude. This finding may be important for several reasons. It has long been assumed that phase retardation in the central macula is the result of the birefringent properties of the HFL (20), but delineation of the HFL boundaries and determination of its thickness are challenging due to its relative optical transparency. Adaptations of existing techniques, such as directional OCT, overcome this constraint and improve optical contrast of the HFL, allowing for improved quantification of its thickness. Here, it is shown that the thickness and phase retardation signals are both minimal centrally, increase proportionally with increasing eccentricity until reaching a maximum, and decline at greater eccentricities. Although this study does not establish causation, the strong correlation between phase retardation distribution and HFL thickness in the central macula provides supporting evidence that the macular phase retardation signal results primarily from the birefringent HFL in healthy, normal subjects.

These results may have considerable clinical implications. The closely matched HFL thickness distribution, centered on the fovea, and phase retardation distribution support previous assumptions that the center of the macular cross corresponds closely with the foveal center, which can be used for foveal localization in *en face* SLP imaging. The correlation between HFL thickness and phase retardation amplitude may also be an optical signature for density or for integrity changes to photoreceptor axons in the central macula. The source of the HFL signal from these two imaging techniques is thought to occur through different mechanisms. Directional OCT uses the angularly dependent scattering properties of the HFL to measure its thickness, and SLP relies on the birefringent properties of the microtubules in the HFL photoreceptor axons. Although directional OCT can be used to quantify HFL thickness, it likely cannot ascertain the components of and the integrity of structures within the HFL. Seemingly counterintuitive to the reduction in phase retardation in the central macula with normal aging (12), histological studies indicate the HFL thickens with age, while other cell layers simultaneously thin (5). This decrease in phase retardation is consistent with high resolution imaging (21) and with histological studies of photoreceptor density (22), indicating a gradual decrease in cone photoreceptor density associated with normal aging. Although they may seem mutually exclusive, it is possible for the HFL to thicken while foveal phase retardation wanes with age. This paradoxical relationship may represent the diversity of cells found in the HFL. Increased HFL thickness found in histological studies is not thought to be related to changes in cone photoreceptor density, but instead attributable to an increased volume of Muller cells, which are present in response to numerous acute and chronic insults as well as to normal aging stresses (5). While contributing to the increased thickness, Muller cells are not thought to possess the same birefringent properties as the photoreceptor axons in the HFL, and are therefore unlikely to contribute to the phase retardation signal in SLP imaging. The thickening of the HFL with reduced phase retardation may occur as a result of declining photoreceptor density or reduced viability of those photoreceptors, making phase retardation a sensitive marker for subtle aging changes.

Beyond aging, the combined use of retinal layer thickness with phase retardation in birefringent retinal structures shows promise in providing insight into early neurodegenerative changes in sight-threatening disease. Examples of the sensitivity of polarization sensitive biomarkers is shown in primate models of glaucoma, where a decrease in the phase retardation of the nerve fiber layer (NFL) precedes thickness changes of the NFL on OCT (23). Histological examination of the NFL in these animals demonstrates residual axonal structure, but in a highly degenerated state. This degeneration results in reduced birefringence, even though the degenerated structures initially occupy the same volume in the retina and NFL thickness is not affected by early degenerative changes. Because the phase retardation signal in both the HFL and the NFL is believed to originate from microtubules in cell axons (10, 24), extrapolating from this animal model suggests that retinal layer thickness and phase retardation could be used similarly for early detection of retinal axonal compromise in the central macula. Identification of retinal cell compromise would be useful in instances where loss of axons or axonal integrity of the central cone and HFL could precede retinal layer thickness changes or when OCT changes are seemingly inconsistent with functional loss, such as Alzheimer's disease (25) and traumatic brain injury (26).

### Variability in Measurements of the Henle Fiber Layer

#### Scanning Laser Polarimetry

Two corneal compensation settings within the same device were used to measure phase retardation of the central macula. As a result, the performance of the two settings could be compared. A fixed compensator was used on the first cohort. This setting can only partially compensate for the polarization properties of the cornea when acquiring retinal images. A variable compensator was used on the second cohort. This setting can more fully compensates for the polarization properties of the cornea compared to the fixed-compensation. Although the two cohorts contained healthy subjects with similar demographic characteristics, the images acquired using fixed anterior segment compensation exhibited significantly greater average pixel intensity and significantly more variability across individuals at all eccentricities than the images acquired using the variable anterior segment compensation. These differences were expected and are likely the result of residual uncompensated anterior segment birefringence, which can vary considerably across individuals. The variable compensator used on the second cohort produced an annular phase retardation map centered on the fovea. Although the overall average pixel intensity was lower at all eccentricities, compared to the fix compensator, there was less variability across individuals. Both in the first cohort and in the second cohort, variability was greatest at the center of the macula and then tapered in the periphery. This relatively high variability near the central macula was likely due to the relatively weak phase retardation signal at that location, where the HFL is absent or very thin. These findings suggest that variable-compensated SLP should be the preferred technique over fixed-compensated SLP for clinical care and for research.

Despite the difference in variability between the two corneal compensation settings, both generated foveal phase retardation maps that were strongly associated with HFL thickness, which was measured with directional OCT. This fact strengthens the validity of our results, and it also may make our results more generalizable.

An enhanced corneal-compensator is available for SLP as a software upgrade that optimizes imaging by improving the signal-to-noise ratio compared with the variable compensation, alone. Although there is some evidence that enhanced compensator correlates well to other imaging modalities (27) and may be more sensitive to changes in the NFL than other compensation strategies (28), it was not utilized in the present study. Future work could correlate its performance with HFL thickness.

#### Directional Optical Coherence Tomography

Even though the HFL thickness profiles of the two groups shared a similar shape, the first cohort exhibited a significantly thicker HFL than the second cohort. Also, there was a wide range of *R*^2^-values in both groups when correlating HFL thickness to pixel intensity. Image quality is a possible cause of the variability between cohorts and of the large range of correlation coefficients. Directional OCT data from three subjects in the second cohort were removed from analysis due to poor image quality metrics. Images from several of the remaining subjects did not cross the threshold for removal from the study but were nevertheless not of ideal quality because the imaging beam of the OCT was not offset enough in the pupil to clearly delineate the HFL from surrounding tissues. Manually segmenting the HFL on a poor-quality OCT images, even those with sufficiently high decibel quality indicators, likely leads to variable thickness measurements, even for well-trained and experienced researchers, because the boarders of the HFL are less defined than in high-quality images.

There are several possible explanations for this difference in subjective image quality. First, the second cohort was larger than the first and contained individuals with small pupils (even after dilation) and deep-set globes. These physical attributes made obtaining high-quality offset OCT images a challenge in some cases. Second, there were different operators of the OCT instrument for the two cohorts, which likely contributed to differences between the two cohorts in how much the imaging beam was offset in the pupil. A third possible explanation is that different individuals segmented the HFL for each cohort. The effect of this variable may be small, however, because previous work has shown manual segmentation of the HFL to be highly repeatable (9).

The development of quality-control software that indicates to the OCT operator when the HFL is of sufficient quality would help eliminate the issue of a poorly delineated HFL in an otherwise high-decibel image. In addition, the creation of software to segment the HFL would likely help mollify the problem of directional OCT being very sensitive to image quality.

### Study Limitations

#### Scanning Laser Polarimetry

A limitation to SLP imaging is that light returning to the system comes from the entire depth of the retina and cannot be axially segmented. While this constraint does not pose a problem for producing a macular cross pattern or a macular annulus pattern in normal individuals, phase retardation changes associated with pathology (29, 30) are difficult to differentiate from overlying or adjacent retina. The combined use of polarized light and cross sectional imaging in polarization sensitive OCT (PS-OCT) can overcome some of these limitations for quantifying HFL phase retardation (31–33), but these systems are expensive, require considerable technical expertise, and are not widely available outside of research institutions.

#### Directional Optical Coherence Tomography

Although directional OCT can visualize the HFL in cross section, it has limitations that currently curb its clinical utility. First the ability, or lack thereof, to quickly and easily collect HFL thickness measurements in multiple meridians. HFL visualization is ideal when the entry position of the imaging beam is along the retinal axis of interest; therefore, imaging in multiple meridians requires technical skill and at least two images, one from each opposite offset positions, along a common meridian. This constraint limited the measurements made in this study to the horizontal and vertical meridians and forced the use of average thicknesses across these meridians as a representative sample of the central macula.

Second, there is no normative database available for HFL thickness. This study represents an initial step in assessing the fovea for changes that could cause alterations to the integrity of the HFL without necessarily impacting the HFL thickness. The full potential of this approach cannot be realized, however, until normative databases of HFL thickness and phase retardation are established.

## Conclusion

This study established a strong and positive association between HFL thickness and phase retardation amplitude in the central macula. It also demonstrated that fixed-compensated SLP produces more varied pixel intensity signals than variable-compensated SLP. With future refinements, the combined use of directional OCT and SLP or other polarization-sensitive imaging methods may be able to detect early changes to photoreceptor integrity in the central macula as a harbinger to ocular or to neurological disease.

## Data Availability Statement

The raw data supporting the conclusions of this article will be made available by the authors, without undue reservation.

## Ethics Statement

The studies involving human participants were reviewed and approved by the Institutional Review Board in Biomedical Sciences at the Ohio State University. The patients/participants provided their written informed consent to participate in this study.

## Author Contributions

PY: study design, data collection, data processing, data analysis, and manuscript writing. MC: study design, data collection, data analysis, and manuscript writing. KR: data collection, data processing, and manuscript writing. DM and ES-G: data collection, data processing, and manuscript review. MO, AZ, and AH: study design and manuscript writing. DV: research question, study design, data processing, data analysis, and manuscript writing. All authors contributed to the article and approved the submitted version.

## Funding

This study was provided by NEI T35 EY007151, by a Career Development Award from the American Academy of Optometry (DV), and by a Lois Hagelberger Huebner Young Investigator Award from the Ohio Lions Eye Research Foundation (PY). PY also received financial support from NEI L30 EY024749 during the study.

## Conflict of Interest

The authors declare that the research was conducted in the absence of any commercial or financial relationships that could be construed as a potential conflict of interest.

## Publisher's Note

All claims expressed in this article are solely those of the authors and do not necessarily represent those of their affiliated organizations, or those of the publisher, the editors and the reviewers. Any product that may be evaluated in this article, or claim that may be made by its manufacturer, is not guaranteed or endorsed by the publisher.
