# Supplementary material for: Foveal Phase Retardation Correlates With Optically Measured Henle Fiber Layer Thickness
Source: Front Med (Lausanne). 2022 Apr 15;9:846738. doi: 10.3389/fmed.2022.846738 (PMC9051020; doi:10.3389/fmed.2022.846738)
Supplement: Supplementary file 1 [file Data_Sheet_1.DOCX]

Supplementary Material


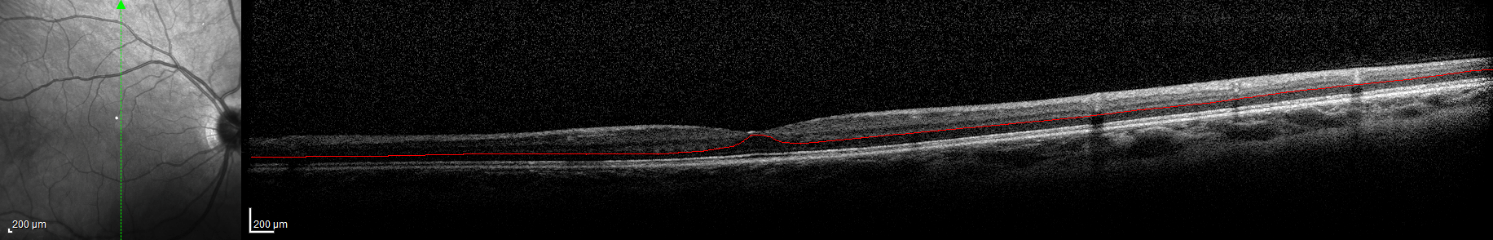


**Figure S1**. Example of a directional optical coherence tomography (OCT) image with a low quality score (18 dB). The dark shadow in the inferior portion of the *en face* infrared image on the left corresponds with the weak signal in the left portion of the cross-sectional OCT scan on the right. Measurements of Henle fiber layer thickness from this subject in the second cohort were not included for analysis.


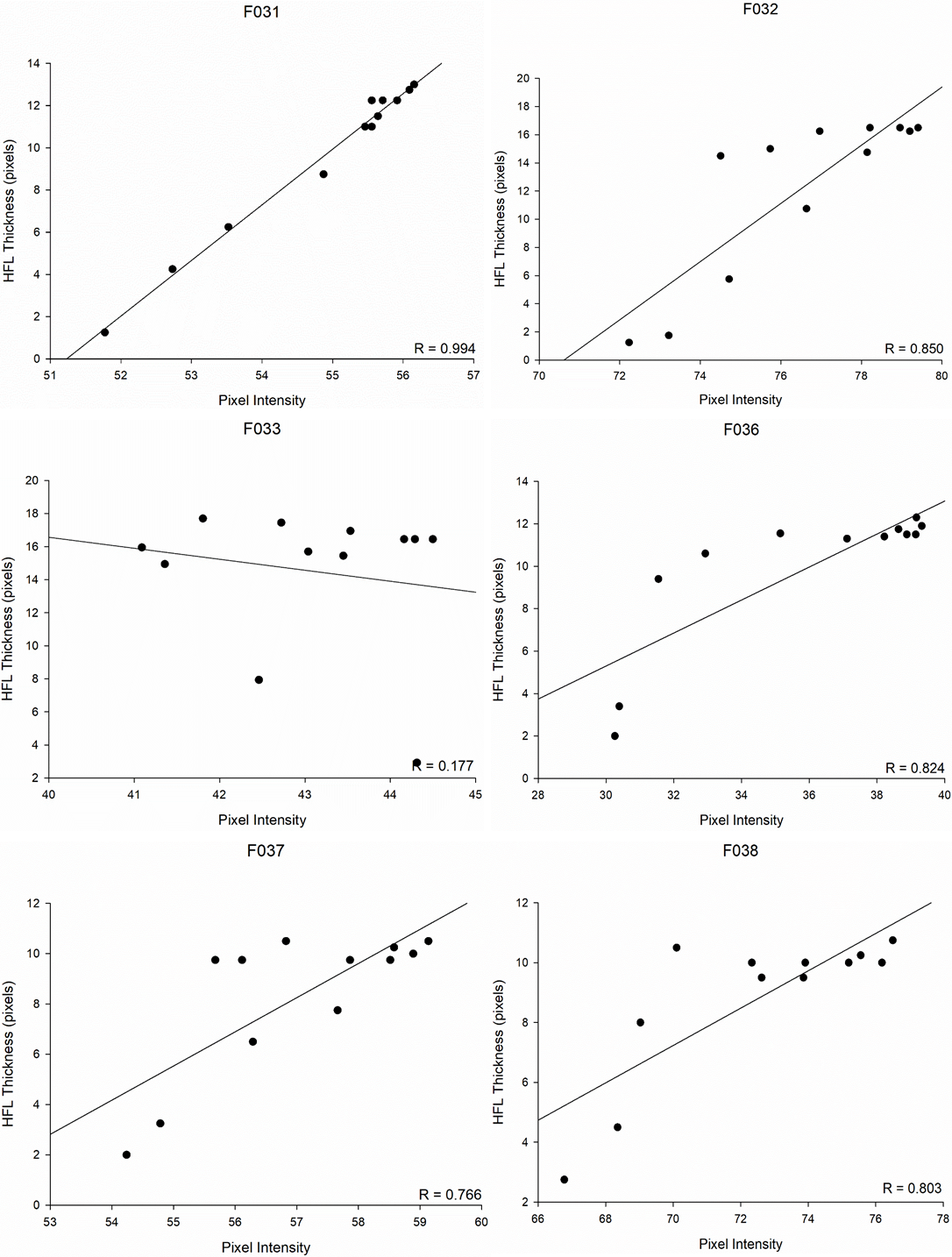


A

B

C

D

F

E


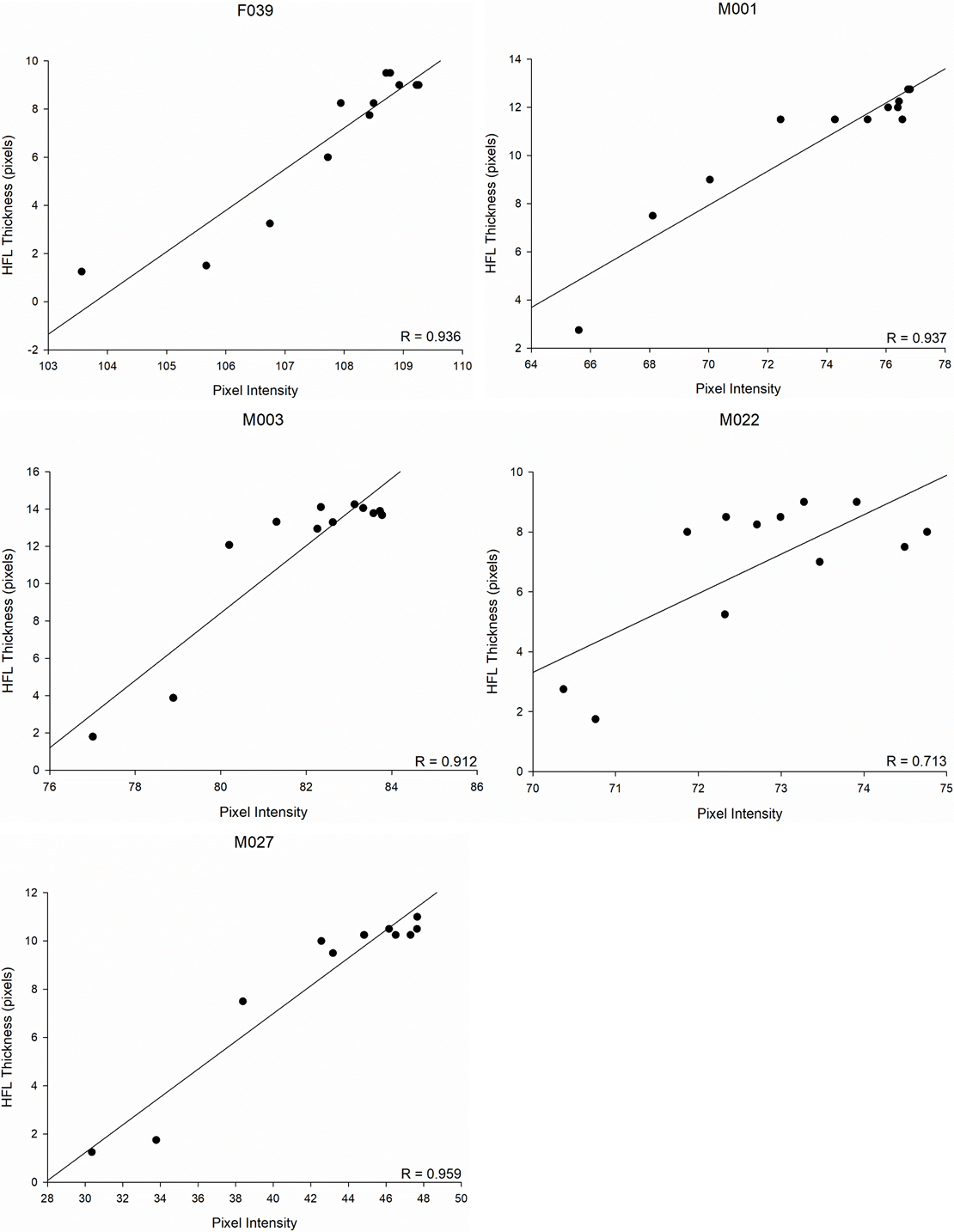


G

H

I

J

K

**Figure S2**. Correlations between Henle fiber layer (HFL) thickness and pixel intensity for individual subjects in the first cohort (A-K). Phase retardation was measured with a fixed-compensated scanning laser polarimeter. R is the correlation coefficient.


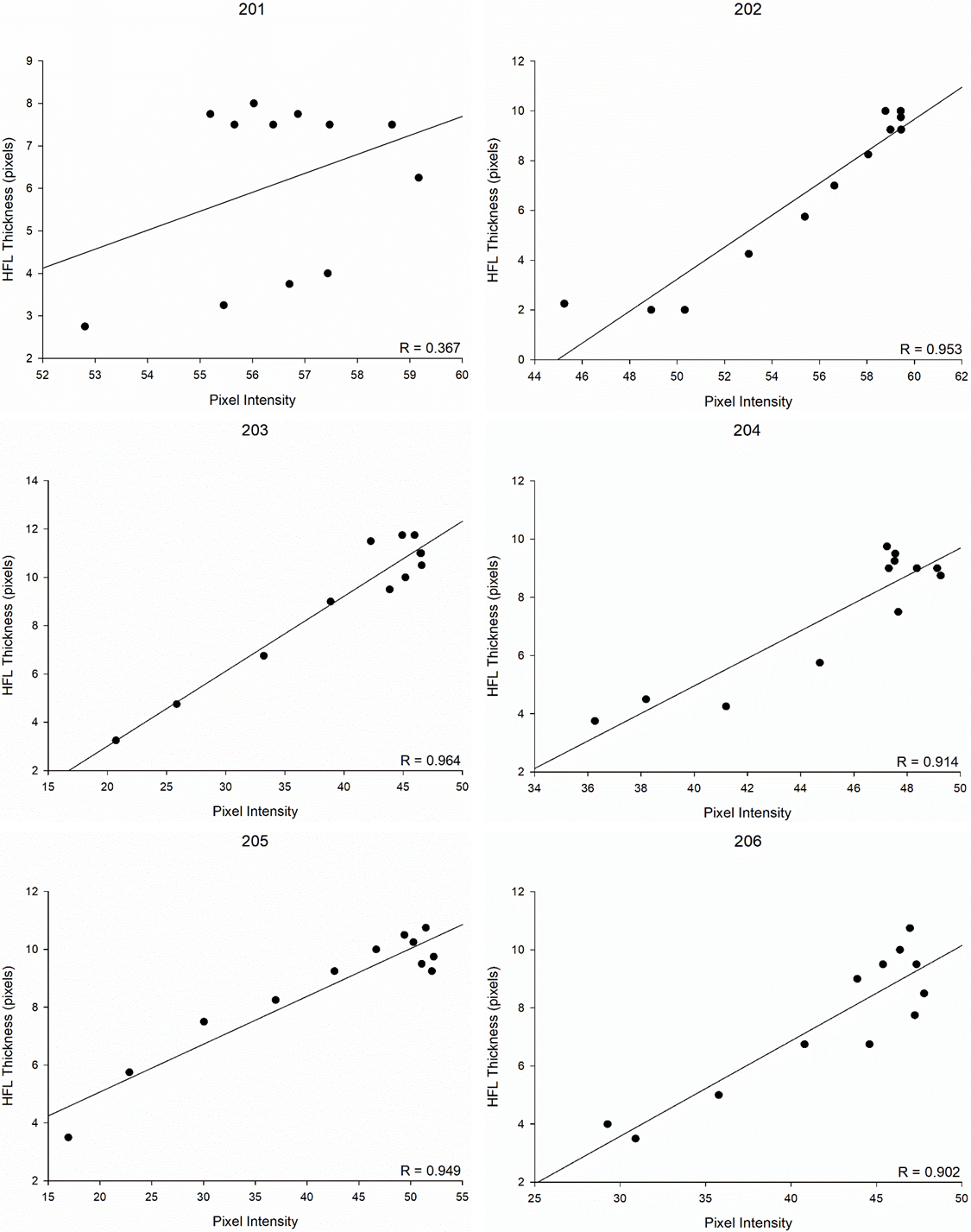


B

A

D

C

E

F


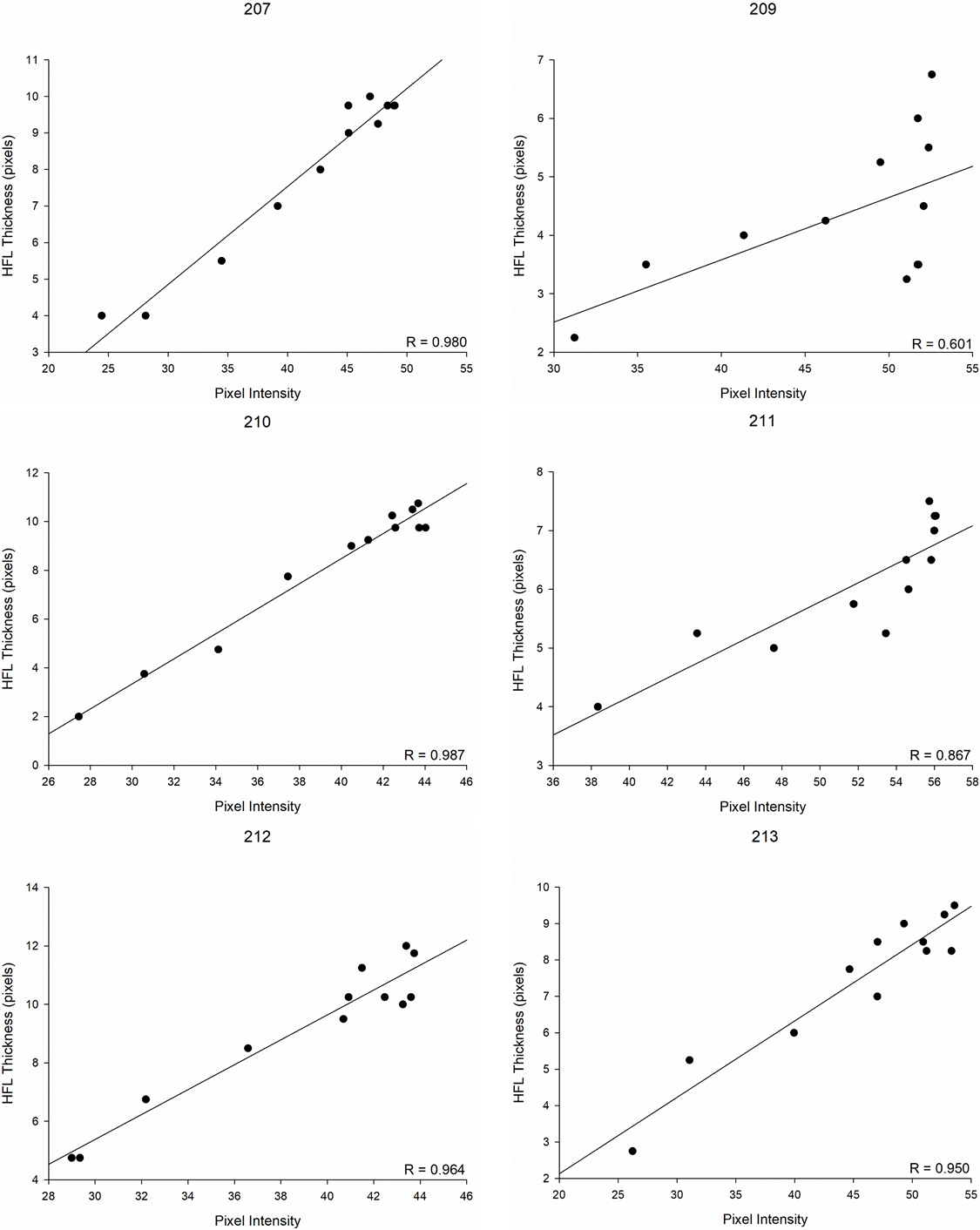


G

H

J

I

L

K


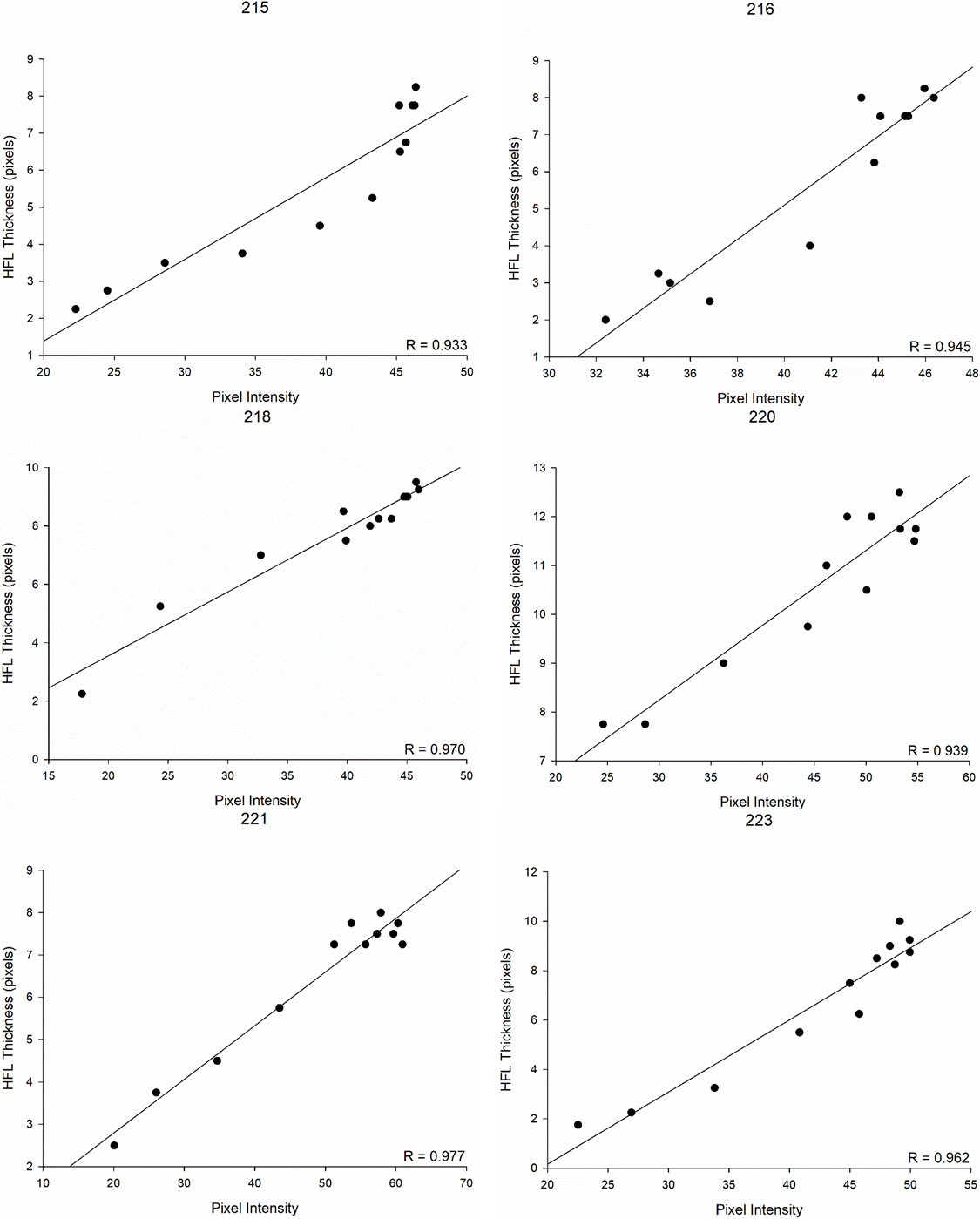


N

M

P

O

R

Q


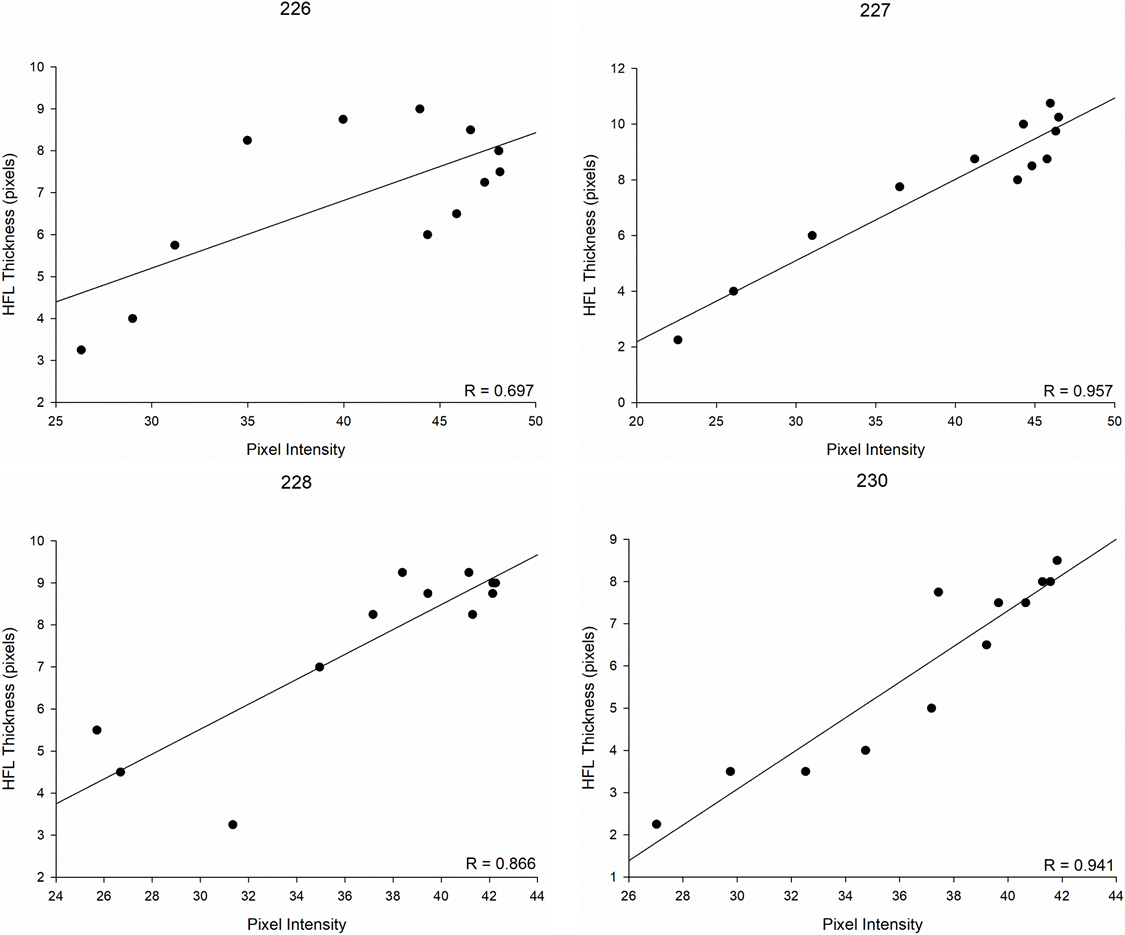


S

T

U

V

**Figure S3**. Correlations between Henle fiber layer (HFL) thickness and pixel intensity for individual subjects in the second cohort (A-V). Phase retardation was measured with a variable-compensated scanning laser polarimeter. R is the correlation coefficient.
